# Targeted regulation of adipose tissue macrophages from an immunometabolic perspective: a novel therapeutic approach for obesity-related metabolic disorders

**DOI:** 10.3389/fimmu.2026.1803488

**Published:** 2026-04-13

**Authors:** Chen Jia, Enfang Wu, Chenjia Zhu, Xiyuan Guan, Yuqi Cheng, Feng Zhao, Xin Jin, Dan Cao, Xueyu Li

**Affiliations:** 1 Department of Disease Prevention and Control, General Hospital of Northern Theater Command, Shenyang, China; 2 General Hospital of Northern Theater Command, Shenyang, Liaoning, China; 3 The Forth Clinical College of China Medical University, Shenyang, China; 4 Shengjing Hospital of China Medical University, Shenyang, China

**Keywords:** adipose tissue macrophages, extracellular vesicles, immunometabolism, metabolic reprogramming, nanotechnology, probiotic extracellular vesicles

## Abstract

The global prevalence of obesity and its associated metabolic syndromes, characterized by chronic inflammation and metabolic disorders, poses a major health threat worldwide. This makes it urgent to gain a deeper understanding of its pathogenesis and to develop new therapeutic strategies. Adipose tissue macrophages (ATMs), as central regulators of the adipose tissue immune microenvironment, exhibit functional polarization closely linked to obesity-associated chronic low-grade inflammation and insulin resistance. This review systematically elucidates the mechanisms of metabolic reprogramming in adipose tissue microenvironment under obesity, focusing on how profound alterations in their glucose, lipid, and amino acid metabolic networks drive their shift toward a pro-inflammatory phenotype. Building on this, we review the mechanisms of action and latest research advances in emerging therapeutic strategies, including mitochondrial-targeted interventions, extracellular vesicle (EV)-mediated molecular delivery, probiotic/prebiotic modulation, probiotic extracellular vesicles, and nanotechnology-enabled precision interventions. Finally, this review outlines the challenges and future directions for treating obesity-related diseases by precisely regulating the EV-ATMs metabolic axis.

## 1 Introduction

The prevalence of obesity and its associated chronic diseases continue to rise globally, affecting over one billion people worldwide and imposing significant socioeconomic burdens (1, 2). It triggers multiple pathological conditions, such as type 2 diabetes, metabolic-associated fatty liver disease, and cardiovascular and neurological disorders (3). Although bariatric surgery offers long-term weight loss solutions for severely obese patients, its invasiveness, risks, and side effects limit widespread adoption, which creates an urgent need for new therapies that control weight effectively while also improving metabolic health (4). Macrophages are essential for maintaining tissue homeostasis and metabolic balance under physiological conditions. In the obese state, macrophages upregulate aerobic glycolysis and downregulate oxidative phosphorylation (OXPHOS) and fatty acid oxidation (FAO), which promotes polarization towards an inflammatory phenotype and immune metabolic imbalance, leading to immune homeostasis disruption and promoting the production of inflammatory cytokines (5). These unique metabolic pathways are the fundamental characteristics of the functional differentiation of macrophages. It is reported that the role of macrophages in obesity is linked to macrophage-mediated metabolic inflammation, manifested as immune cell infiltration and persistent macrophage activation, which are key factors contributing to obesity-related conditions (6). Emerging evidence indicates that Adipose tissue macrophages (ATMs) are key participants in the development of obesity and associated metabolic inflammation (7). Therefore, understanding how adipocytes interact with the immune microenvironment and targeting dysregulated adipose tissue macrophages have become highly promising new strategies for reversing obesity-related metabolic diseases. This review aims to systematically elucidate the latest mechanistic advances in immune-metabolic dysregulation within adipose tissue during obesity progression. It focuses on dissecting the core role of macrophages and the regulatory influence of the gut microbiome, while prospectively exploring innovative therapeutic strategies based on the concepts of “metabolic-immune microenvironment remodeling” and “precision intervention.”

## 2 Origins of ATMs and polarization

**Figure 1:**
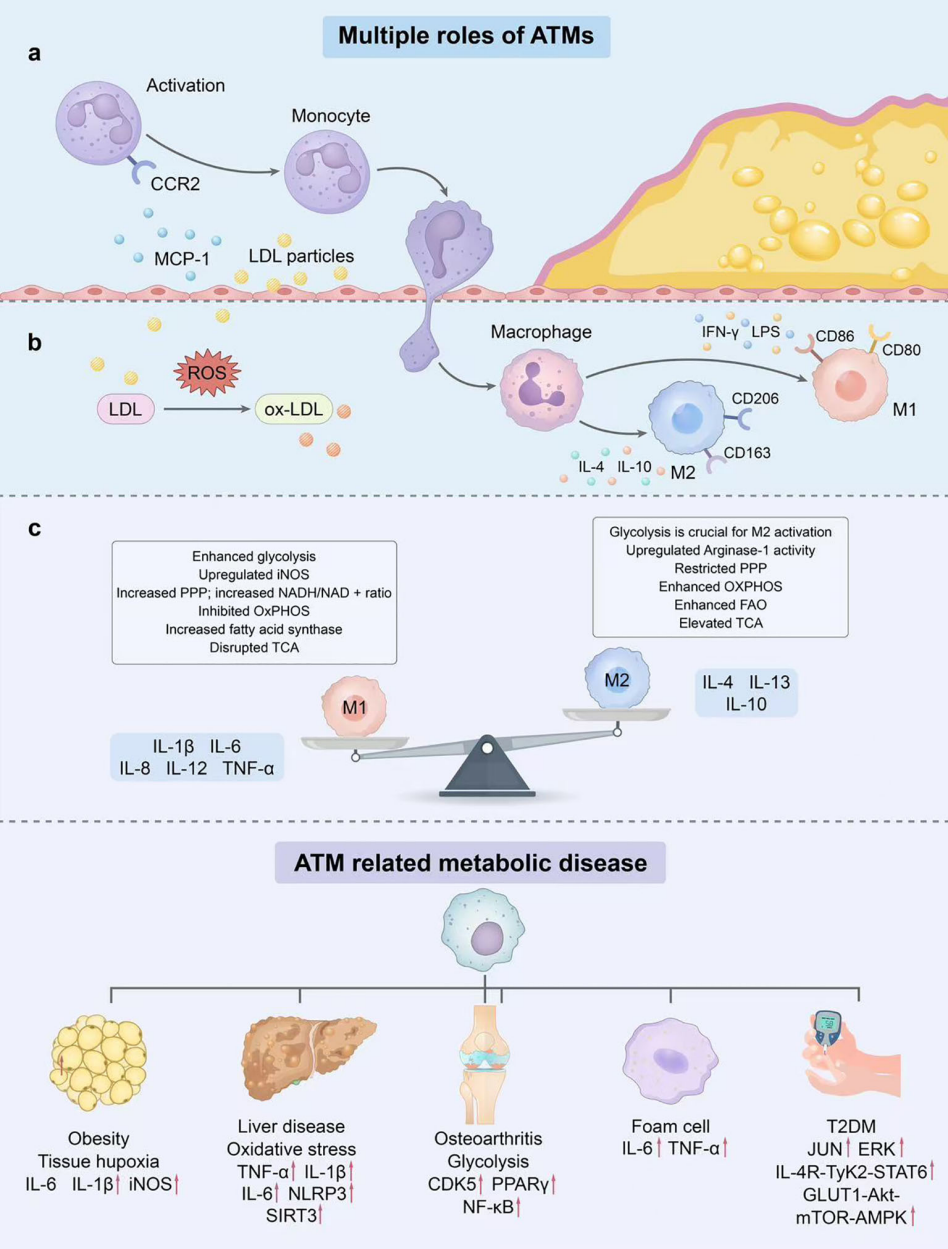
ATMs drive obesity-related metabolic diseases via recruitment, polarization, and metabolic reprogramming. **(a)** Monocyte recruitment and polarization: Obesity-induced MCP-1 binds CCR2 to recruit monocytes. Pro-inflammatory signals (IFN-γ, LPS) induce M1 polarization (CD80/CD86^+^) with pro-inflammatory cytokines (IL-1β, TNF-α, IL-6), while anti-inflammatory cues (IL-4, IL-13) promote M2 polarization (CD163/CD206^+^) and IL-10 secretion. Ox-LDL accelerates foam cell formation and inflammation. **(b)** Core ATM metabolism: M1 macrophages rely on aerobic glycolysis (elevated ROS), whereas M2 macrophages depend on oxidative phosphorylation OXPHOS and FAO. **(c)** Disease regulation network: ATMs modulate type 2 diabetes, non-alcoholic fatty liver disease, and osteoarthritis via cytokines and signaling pathways. ATMs, Adipose tissue macrophages; MCP-1, Monocyte Chemoattractant Protein-1; CCR2, C-C motif chemokine receptor 2; IFN-γ, Interferon-gamma; LPS, Lipopolysaccharide; IL-1β, Interleukin - 1β; TNF-α, Tumor necrosis factor-alpha; IL-6, Interleukin-6; IL-4, Interleukin-4; IL-13, Interleukin-13; Ox-LDL, Oxidized low-density lipoprotein; ROS, Reactive oxygen species; OXPHOS, Oxidative phosphorylation; FAO, Fatty Acid Oxidation.

White Adipose tissue (WAT) functions as a highly active endocrine and immune organ, and its dysregulation constitutes a core feature of obesity-related metabolic diseases. Among all immune cells in WAT, macrophages constitute the largest population, accounting for 50% of the adipose stromal vascular fraction in obese individuals. These macrophages have been demonstrated to play a pivotal role in WAT remodeling and are associated with metabolic disease risk (8). As the most abundant immune cells in adipose tissue, ATMs are dynamically regulated in obesity: overexpression of Monocyte Chemoattractant Protein 1 and CCL2/C-C chemokine receptor 2 (CCR2) in WAT promotes macrophage accumulation (9, 10). Specifically, activation of the CCR2 pathway mediates the infiltration of circulating monocytes into obese WAT, further expanding the ATMs pool (11). ATMs exhibit high heterogeneity and plasticity, with its phenotype and function dynamically regulated by local microenvironmental signals (12). Functionally, macrophages can be categorized into two major types: M1 and M2 macrophages. M1 macrophages are typically considered pro-inflammatory, as they secrete IL-1β, IL-6, IL-8, IL-12, and TNF-α and play a crucial role in tissue injury (13). Conversely, M2 macrophages are generally anti-inflammatory, secreting IL-4, IL-13, and IL-10, and are associated with wound healing, inflammation resolution, clearance of cellular debris, regulation of proliferation, and Extracellular Matrix remodeling (14). In humans, M1 macrophages tend to express surface markers such as CD11c, CD14, and CD40, while M2 macrophages most commonly express CD163 and CD206 (15). Although this M1/M2 macrophage paradigm was initially a useful model, recent advances suggest macrophages may exhibit more complex phenotypic diversity and a broader range of activation states. For specific phenotypes and their polarization features, please refer to **Table 1**. The integrated mechanism by which ATMs recruitment, polarization, and metabolic reprogramming collectively drive obesity-related metabolic diseases is depicted in **Figure 1**. This diversity suggests that in obesity-related metabolic complications, different macrophage subtypes may interact, sometimes working together, sometimes opposing each other, to influence disease development. As an endocrine organ, WAT secretes numerous adipokines including TNF-α, IL-6, adiponectin, leptin, fatty acid-binding protein 4 (FABP4), and granulocyte-macrophage colony-stimulating factor (16). Beyond these secreted factors, intracellular signaling hubs also dictate ATMs fate. For instance, the endoplasmic reticulum stress sensor inositol-requiring enzyme 1αhas been identified as a key regulatory node: its activation promotes the shift of ATMs toward a pro-inflammatory M1-like phenotype while suppressing the anti-inflammatory M2-like subpopulation, thereby exacerbating metabolic dysfunction (17). Notably, in both human and mouse models, increased ATMs numbers and their polarization toward a pro-inflammatory M1-like phenotype show significant positive correlations with obesity severity and metabolic dysfunction (18). Under the condition of obesity, the nutritional imbalance, accumulation of inflammatory factors and disorder of intercellular signals in the WAT microenvironment directly trigger profound reprogramming of the sugar, lipid and amino acid metabolic networks of ATMs. This metabolic reprogramming not only provides energy and material basis for the immune activation of ATMs, but also, by regulating the activation and silencing of inflammatory signaling pathways, becomes the core molecular mechanism that drives the shift of ATMs towards the pro-inflammatory M1-like phenotype and inhibits the anti-inflammatory M2 phenotype. Therefore, analyzing the metabolic reprogramming rules of ATMs in the obese microenvironment is a key prerequisite for clarifying their polarization regulatory mechanism and finding targeted intervention targets.

**Table 1:** Summary of macrophage subtypes induced by different stimulating factors and their phenotypic markers.

ATMs typing	Phenotype	Mouse or human	Function	Reference
MICAM	CD11c+CD40+CD14+	Both	direct inflammatory changes in adipose tissue	(19)
M2AAM	CD163+CD206+CD209+	Both	Anti-inflammatory, maintain WAT homeostasis, improve insulin sensitivity	(20)
MME	CD36, ABCA-1	Both	Metabolic activation associated with insulin resistance state	(21)
LAM	Trem2, CD9	Both	Macrophage remodeling, LAM cell formation, and coronary structure assembly during obesity	(22)
M4	CD68, S100A8	Mouse	Related to the instability of the plaque	(23)
MOX	CD11c、CD206、CD163, HO-1	Mouse	maintain redox homeostasis or promote inflammation	(24)

M2AAM, M2 anti-inflammatory adipose tissue macrophages; M1CAM, M1 classically activated adipose tissue macrophages; LAM, lipid-associated macrophages; MOX, Metabolically activated macrophages; MMe, metabolically activated macrophages; HO-1, Heme Oxygenase-1.

## 3 Metabolic remodeling of ATMs in the obesity microenvironment

### 3.1 Glucose metabolism

**Figure 2:**
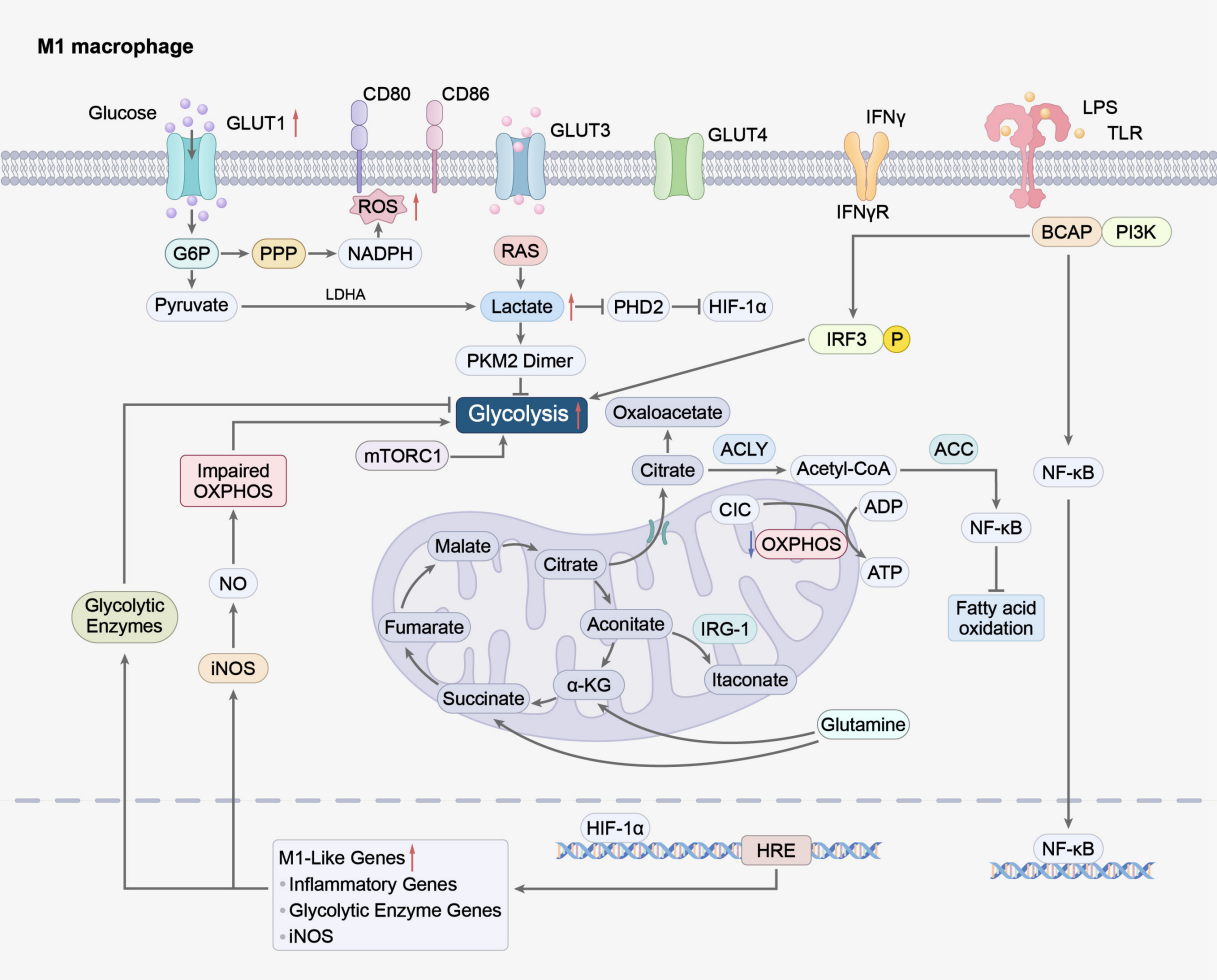
M1 macrophages undergo metabolic reprogramming dominated by aerobic glycolysis upon stimulation with pro-inflammatory signals (e.g., IFN-γ, LPS), driving pro-inflammatory phenotype formation. Glucose is transported into cells via GLUT1 and GLUT3, and metabolized through glycolysis to produce pyruvate, which is further converted to lactate by LDHA. Lactate stabilizes HIF-1α by inhibiting PHD2, forming a positive feedback loop to enhance glycolytic enzyme expression (via PKM2 dimer nuclear translocation and HIF-1α activation). Glucose metabolism also activates the PPP to generate NADPH, promoting ROS and NO production. Impaired TCA cycle leads to accumulation of metabolites which activate pro-inflammatory signaling pathways (NF-κB, IRF3). These metabolic and signaling events synergistically upregulate pro-inflammatory gene expression, consolidating the M1 pro-inflammatory phenotype. IFN-γ, Interferon-gamma; LPS, Lipopolysaccharide; LDHA, Lactate Dehydrogenase A; HIF-1α, Hypoxia inducible factor 1 subunit alpha; PHD2, Prolyl Hydroxylase; PKM2, Pyruvate Kinase M2; PPP, Pentose phosphate pathway; NADPH, Nicotinamide Adenine Dinucleotide Phosphate Hydrogen; ROS, Reactive oxygen species; NF-κB, Nuclear factor kappa-B; IRF3, Interferon Regulatory Factor 3.

In the adipose tissue microenvironment, ATMs undergo profound metabolic reprogramming, with the shift from OXPHOS to aerobic glycolysis serving as a core metabolic event initiating and sustaining its proinflammatory phenotype (25). This metabolic switch not only rewires the energy production mode of ATMs but also drives the activation of redox and inflammatory signaling pathways, forming a metabolic-inflammation positive feedback loop closely linked to obesity-related metabolic disorders. In ATMs, LPS binds to its receptor TLR4, triggering downstream activation of the NF-κB and PI3K/AKT signaling pathways. Specifically, NF-κB translocates to the nucleus and directly binds to the GLUT1 (SLC2A1) promoter at the -350 to -280 bp region (25, 26). GLUT1 serves as the primary rate-limiting mediator for glucose uptake in M1-type ATMs (27), and its expression—along with that of hypoxia-inducible factor-1α (HIF-1α) is significantly upregulated in visceral WAT-derived ATMs from obese human individuals (28). This upregulation ensures sustained glucose acquisition to support pro-inflammatory mediator synthesis (27, 29, 30). Enhanced glucose influx further activates the pentose phosphate pathway (PPP) while accelerating glycolytic flux, where PPP mediates increased NADPH production to drive the generation of reactive oxygen species (ROS) and nitric oxide (NO), key mediators of ATM inflammatory activation (27) Glucose metabolism stimulate G6PD in macrophages through a multi-level regulatory process. The nuclear translocation of the key metabolic enzyme pyruvate kinase M2 subunit (PKM2) and the stabilization of HIF-1α jointly transcriptionally upregulate multiple glycolytic enzyme genes, significantly enhancing glycolytic flux (31). HIF-1α itself is directly regulated by lactate, the product of glycolysis. Physiological lactate concentrations competitively inhibit prolyl hydroxylase 2 (PHD2), preventing HIF-1α degradation and forming an amplified feedforward loop (32). The specific metabolic reprogramming pathway of M1 type ATM, which mainly relies on aerobic glycolysis, is shown in **Figure 2**. Among them, the lactate - HIF-1α positive feedback loop is the core regulatory node. Moreover, increased glycolytic flux is directly linked to the production of pro-inflammatory cytokines (33). For instance, fructose enhances LPS-induced inflammatory cytokine synthesis by boosting mTORC1 activity. This metabolic reprogramming culminates in pyruvate accumulation, diverting it into the tricarboxylic acid (TCA) cycle and *de novo* lipogenesis pathways, laying the groundwork for lipid accumulation (34). ATM-mediated glucose metabolic reprogramming exhibits high heterogeneity. Transporters like GLUT3 may participate in regulating alternative activation through glucose-independent functions, RAS-mediated actions to effectively regulate endothelial cell proliferation and IL-4/STAT6 activation, thereby modulating alternative macrophage polarization and function (35). The metabolic state of ATMs can also remotely influence systemic metabolic homeostasis via paracrine mechanisms. For instance, ATMs-derived miR-210-3p disrupts systemic insulin sensitivity by suppressing adipocyte GLUT4 expression (36), revealing a direct link between ATMs metabolic status and whole-body metabolic homeostasis.

### 3.2 Lipid metabolism

**Figure 3:**
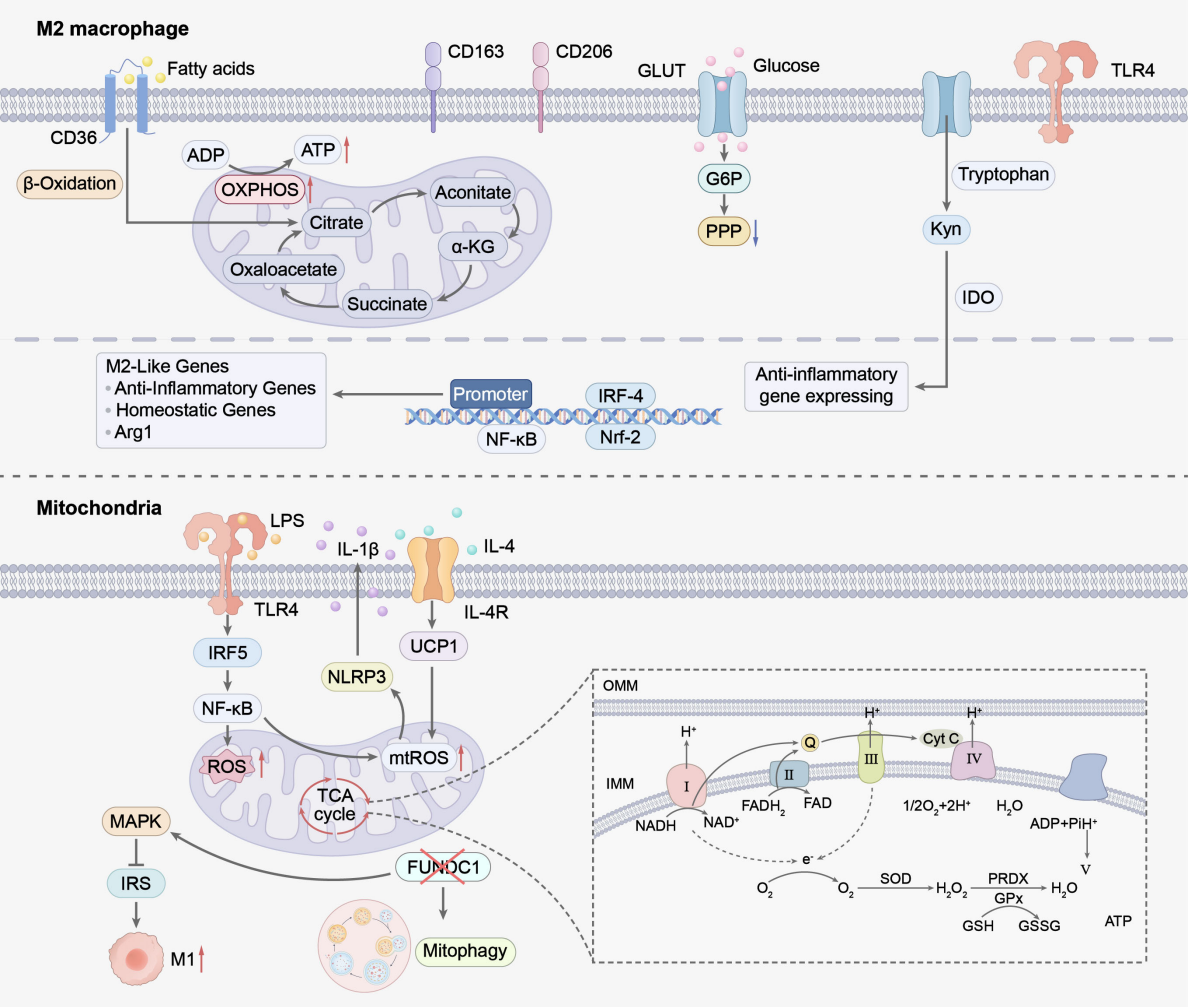
This figure reveals the complex mechanisms of M2-type macrophages in metabolic reprogramming, as well as their crucial role in regulating immune responses.M2 macrophages maintain anti-inflammatory phenotypes through metabolic pathways centered on OXPHOS and FAO. Fatty acids are taken up via CD36 and undergo β-oxidation, while glucose is transported via GLUTs and metabolized to support PPP and TCA cycle activity. The TCA cycle intermediate α-KG modulates epigenetic reprogramming (via Jmjd3) to promote anti-inflammatory gene expression. Tryptophan metabolism by IDO generates Kyn to exert immunosuppressive effects, and itaconate (derived from aconitate via IRG-1) inhibits pro-inflammatory signaling. Anti-inflammatory signals (e.g., IL-4) activate STAT6 and IRF4, driving expression of M2 markers and anti-inflammatory cytokines. Intact mitochondrial function and mitophagy mediated by FUNDC1 prevent mtROS accumulation, while Nrf2 pathway activation enhances antioxidant capacity. UCP1-mediated uncoupling regulates energy metabolism and systemic thermogenesis, collectively sustaining M2 anti-inflammatory and tissue-homeostatic functions. OXPHOS, Oxidative phosphorylation; FAO, Fatty Acid Oxidation; PPP, Pentose phosphate pathway; α-KG, A-Ketoglutaric acid; IDO, Indoleamine-2, 3-Dioxygenase; IRG-1, Immune-responsive gene 1; IL-4, Interleukin-4; STAT6, Signal Transducer And Activator Of Transcription 6; IRF4, Interferon Regulatory Factor 4; FUNDC1, FUN14 domain containing 1; Nrf2, Nuclear factor erythroid 2-related factor 2; UCP1, Uncoupling Protein 1.

ATMs play a central role in maintaining adipose tissue homeostasis. Unlike classical inflammatory activation patterns, ATMs in obesity exhibit unique metabolic reprogramming characterized by marked activation of lipid metabolism pathways (**Figure 3**) (37). In contrast, M2 macrophages primarily rely on OXPHOS and FAO, maintaining an intact and highly active TCA cycle (38). Studies in murine models have revealed that ATP-binding cassette transporter G1(ABCG1) plays a critical role in shaping ATM phenotypes. ABCG1 knockout reduces lipoprotein lipase (LPL) activity, promoting redistribution of saturated fatty acids (SFAs) from membrane phospholipids to lipid droplets. This decreases membrane rigidity and attenuates the pro-inflammatory M1-like phenotype of ATMs, ultimately improving WAT inflammation and insulin resistance in obese mice. These findings suggest that targeting membrane lipid composition may represent a novel strategy for treating obesity-related metabolic diseases. In adipocytes, elevated intracellular monoglyceride (MAG) levels activate peroxisome proliferator-activated receptors (PPARs), while MAG secreted by adipocytes suppresses macrophage polarization toward an inflammatory phenotype and promotes precursor adipocyte differentiation (39). Furthermore, free fatty acids (FFAs) activate signal transducer and activator of transcription 3 (STAT3) in macrophages, thereby inducing M1 cytokine production (40). In obesity, elevated circulating nutritional fatty acids exacerbate metabolic inflammation by activating Toll-like receptor 4 (TLR4) signaling in both adipocytes and macrophages (41). Notably, obesity activates a lysosome-dependent lipid metabolism pathway in ATMs independent of classical inflammatory activation pathways, as demonstrated in murine models (42). Within this pathway, complex lipids are broken down by lysosomal acid lipase into free cholesterol and FFAs. Subsequently, FC and FFAs are transported to the endoplasmic reticulum, where acyl-CoA: cholesterol acyltransferase-1 esterifies cytotoxic FC into cholesterol esters, while diacylglycerol acyltransferase converts FFAs into the storage form triacylglycerol (TAG) (43). This lipid remodeling process may protect cells from lipotoxicity. Regarding inflammatory responses, lipid accumulation in M1 macrophages drives proinflammatory cytokine and ROS production, thereby exacerbating inflammation and tissue damage. Studies indicate that inhibiting mitochondrial ROS production reduces NF-κB activation and suppresses proinflammatory cytokine expression induced by oxidized low-density lipoprotein (oxLDL) (44), suggesting that lipid-mediated oxidative stress plays a crucial role in ATM inflammation regulation. Emerging evidence has identified additional molecular nodes linking lipid metabolism to ATM function. FABP4, a cytoplasmic fatty acid chaperone highly expressed in both adipocytes and macrophages, has been implicated in the development of insulin resistance and atherosclerosis. Mechanistic studies in murine macrophages demonstrate that FABP4 triggers ubiquitination and subsequent proteasomal degradation of peroxisome proliferator-activated receptor γ (PPARγ), a master transcriptional regulator of adipogenesis and insulin sensitivity (45). By downregulating PPARγ, FABP4 disrupts metabolic homeostasis in both adipocytes and macrophages, thereby exacerbating local and systemic inflammation. The cholesterol metabolism regulator SCAP (SREBP cleavage-activating protein) exhibits tissue-specific functions in inflammation (46). In murine macrophages, SCAP overexpression promotes the translocation of both SCAP itself and the NLRP3 inflammasome to the Golgi apparatus, enhancing NLRP3 inflammasome activation and accelerating atherosclerosis (47). Conversely, in murine liver and WAT, SCAP attenuates STING–NF-κB signaling activation, thereby ameliorating local inflammation (48). This dual role highlights the complex interplay between cholesterol metabolism and innate immune signaling. The intrinsic lipid metabolic activity of macrophages also directly shapes their immune phenotype. Adipose triglyceride lipase (ATGL) catalyzes the first step of intracellular triglyceride hydrolysis, generating non-esterified fatty acids (49). Interestingly, in murine macrophages, ATGL not only mediates TG lipolysis but also participates in immune regulation. ATGL deficiency in lipid-laden macrophages attenuates the release of the pro-inflammatory cytokine IL-6 while enhancing the anti-inflammatory cytokine IL-10 (50). Further studies confirm that reduced ATGL-mediated lipolysis dampens macrophage inflammatory responses upon activation, accompanied by decreased production of prostaglandin E2 (PGE2) and IL-6 (51). These findings suggest that modulating the intrinsic lipid metabolic flux of macrophages may represent an effective strategy for reshaping their inflammatory phenotype.

Notably, a close crosstalk exists between glucose and lipid metabolism. Citrate, an intermediate of the TCA cycle, serves as a critical link connecting glycolysis to lipid synthesis in M1-like macrophages. Under pro-inflammatory conditions, enhanced glycolytic flux leads to mitochondrial citrate accumulation. Citrate is exported to the cytoplasm via the mitochondrial citrate carrier (CIC) and cleaved by ATP citrate lyase (ACLY) into acetyl-CoA and oxaloacetate (52). Accumulated citrate further activates acetyl-CoA carboxylase (ACC), which converts acetyl-CoA into malonyl-CoA. Malonyl-CoA inhibits carnitine palmitoyltransferase 1 (CPT1), thereby interfering with fatty acid oxidation and promoting lipid accumulation while impairing metabolic flexibility (53). This “glycolysis–citrate export–lipogenesis” axis provides ample substrates for lipid accumulation and inflammatory mediator synthesis in M1 macrophages, explaining why pro-inflammatory M1-like frequently exhibit significant lipid droplet accumulation. Targeting the CIC-ACLY-ACC metabolic axis may therefore represent a dual strategy to simultaneously intervene in both glucose metabolic reprogramming and lipid accumulation in ATMs.

### 3.3 Amino acid metabolism

WAT has emerged as a key regulator of amino acid metabolism, with significant implications for ATMs function and polarization. Glutamate, an abundant amino acid, plays multiple roles in cellular energy regulation, with elevated plasma glutamate levels observed in obese individuals (54). Studies in murine macrophages have shown that glutamine/glutamate metabolism supports anti-inflammatory macrophage polarization via metabolic reprogramming of the TCA cycle (55). During synchronized TCA cycle activity, glutamine is converted to glutamate, which is then converted to α-ketoglutarate(α-KG). Elevated α-KG levels play a prerequisite role in the polarization of anti-inflammatory macrophages, involving fatty acid oxidation and Jmjd3-dependent epigenetic reprogramming of anti-inflammatory genes. A high α-KG-to-succinate ratio supports this polarization mechanism, while a low ratio pushes macrophages toward a pro-inflammatory state (56). This metabolic-epigenetic coupling reveals how amino acid metabolism directly shapes macrophage identity. Glutamine is another abundant amino acid critical for metabolism, immunity, and nitrogen homeostasis (57). Emerging evidence highlights the crucial role of glutamine metabolism in regulating ATM inflammatory status. Studies in human macrophages and adipose tissue samples have revealed that downregulation of glutamine synthetase promotes pro-inflammatory factor expression. Conversely, glutamine supplementation exerts anti-inflammatory effects by inhibiting glycolysis and reducing O-GlcNAcylation levels—a process closely linked to the transcription factor SP1 (58). These findings suggest that maintaining adequate glutamine availability may represent a nutritional strategy to restrain ATM-mediated inflammation. Notably, although glutamine and α-ketoglutarate levels are lower in obesity, elevated succinate levels are associated with pro-inflammatory macrophage polarization in rat models (59), indicating that the balance between multiple metabolites, rather than any single metabolite, dictates macrophage fate. Metabolomic analysis indicates that increased branched-chain amino acids (BCAAs) metabolism is a significantly altered pathway during macrophage polarization both *in vivo* and *in vitro*. Studies in murine macrophages demonstrate that BCAAs reduce IL-6 expression, subsequently attenuating pro-inflammatory functions (60). Interestingly, research in human subjects shows that BCAAs oxidase activity is lower in the adipose tissue of obese, insulin-resistant individuals, where decreased expression of Branched-Chain Amino Acid Transaminase 2 and Branched-Chain α-Keto Acid Dehydrogenase further inhibits anti-inflammatory macrophage polarization (61). This obesity-associated defect in BCAAs catabolism may contribute to sustained adipose tissue inflammation.

### 3.4 Bidirectional metabolic crosstalk between ATMs and WAT

The bidirectional metabolic crosstalk between ATMs and adipocytes is the core mechanism shaping the local immune metabolic microenvironment of WAT, and it is also the key hub linking the dysfunction of adipose tissue with systemic metabolic diseases. M1-ATMs can activate the JNK/NF-κB and JAK/STAT pathways in adipocytes by secreting pro-inflammatory factors such as TNF-α and IL-1β, upregulate the expression of lipolytic enzymes such as HSL and ATGL, and inhibit the activity of Perilipin 1 (62). At the same time, they can block the insulin signaling pathway of IRS1-AKT-PI3K and directly promote excessive lipolysis in adipocytes and damage their lipid storage function (51, 63). M2-type ATMs can reverse the lipolysis by secreting IL-10 and TGF-β, thereby restoring the metabolic homeostasis of adipocytes (64). Meanwhile, ATMs can also regulate the secretion spectrum of adipocyte adipokines. On one hand, they inhibit the release of protective adipokines such as adiponectin and SFRP5, and on the other hand, they upregulate the secretion of pathogenic factors such as FABP4 and RBP4 (65). Among them, FABP4 is degraded by the ubiquitin-proteasome pathway through PPARγ, exacerbating metabolic disorders (45). Adiponectin and SFRP5 can target the liver through the circulatory system via the AMPK pathway to improve hepatic glycolipid metabolism and inhibit the activation of hepatic stellate cells through the Wnt5a pathway (65). RBP4 directly promotes hepatic gluconeogenesis and exacerbates liver inflammation.

Dietary lipids can regulate the uptake of mitochondria by ATMs, and when the uptake function is impaired, adipocytes will release oxidatively damaged mitochondria into the circulation through small EVs (66). These damaged mitochondria will not only exacerbate the oxidative stress and inflammatory activation of ATMs but also transfer to the liver to trigger oxidative damage and inflammatory responses in liver cells. The functional specialization of ATGL in ATMs also participates in the crosstalk regulation between the two, and its expression deficiency can significantly inhibit the release of pro-inflammatory factors from ATMs and reverse the inflammatory microenvironment of adipocytes (67). Moreover, ATMs can further regulate the secretion of adipokines and insulin signaling by releasing EVs carrying miR-27a-5p and FABP4 (68). This multi-dimensional bidirectional regulation disorder ultimately leads to the release of a large amount of FFA and pathogenic signaling molecules into the blood, continuously disrupting the local stability of adipose tissue, and through systemic energy diversion and cross-organ signal transduction, it becomes the core pathological basis for promoting metabolic disorders in peripheral tissues such as the liver and the occurrence and development of diseases such as MASLD. This complex regulatory network is also subject to external regulation by the gut microbiota and its metabolites, thereby forming a broader cross-organ metabolic regulatory system.

## 4 Mitochondrial metabolism of ATMs: the core hub of immunometabolic reprogramming

The metabolic reprogramming of sugar, lipid and amino acids in ATMs is not an independent process, but rather a mutually interrelated and jointly converging process within the functional regulation of mitochondria. Mitochondria is a central hub integrating cellular metabolism and inflammatory signaling (69). The regulatory network underlying mitochondrial metabolism−driven ATM functional reprogramming is illustrated in **Figure 3**. As the primary drivers of OXPHOS, mitochondria generate cellular ATP via the electron transport chain (ETC), a series of protein complexes (Complexes I-IV) embedded in the inner mitochondrial membrane that transfer electrons from reduced substrates to molecular oxygen, creating an electrochemical gradient utilized by Complex V (ATP synthase) to produce ATP (70). In WAT, macrophage immune function is closely connected to their metabolic state, forming a positive-feedback inflammatory cycle. Elevated LPS and free fatty acids in this cycle activate TLR4 on macrophage surfaces, driving classical pro-inflammatory signaling pathways such as IRF5/NF-κB (41). This not only directly promotes the development of insulin resistance (71) but also profoundly reprograms cellular energy metabolism, with the TCA cycle being the core link of this reprogramming. Intermediates of the TCA cycle act as crucial signal hubs in this metabolic remodeling process: succinate accumulates in mitochondria under obese or hypoxic conditions in ATMs, and the accumulated succinate promotes inflammatory responses by stabilizing HIF-1α on the one hand; on the other hand, it is transported to the extracellular space and regulates the functions of adipocytes and immune cells in an autocrine/paracrine manner through its receptor SUCNR1, exacerbating local inflammation and metabolic disorders. In contrast, itaconate exerts important immunomodulatory and protective effects as a key TCA cycle intermediate. Itaconate is one of the most highly upregulated metabolites in inflammatory macrophages, which is produced by the conversion of aconitate via the enzyme immunoresponsive gene 1 (IRG1) as a result of TCA cycle interference (72). As an emerging immunomodulator, itaconate has antibacterial defense and anti-inflammatory effects, and has recently been identified as a suppressor of obesity. Mechanistic studies have shown that itaconate upregulates key proteins involved in fatty acid oxidation and inhibits the expression of adipogenic genes in ATMs; it may also trigger significant metabolic reprogramming by inducing fatty acid oxidation and inhibiting fatty acid synthesis in brown adipose tissue, thereby counteracting the pro-inflammatory and lipogenic metabolic disturbances of ATMs in obesity (73). A central consequence of this metabolic reprogramming is excessive ROS production, particularly mitochondrial ROS (mtROS). Acting as a key signaling molecule, mtROS activates the NLRP3 inflammasome, further amplifying the production of inflammatory mediators like IL-1β release, tightly linking metabolic stress to chronic inflammation (74). Mitochondrial quality control serves as a pivotal regulator of this process.faulty mitophagy exemplified by loss of the mitochondrial receptor FUNDC1 leads to dysfunctional mitochondria and mtROS accumulation, exacerbating M1 macrophage infiltration and diet-induced obesity (75, 76). Conversely, the APPL1-Rab5 signaling axis effectively clears damaged mitochondria by enhancing early endosome-dependent mitophagy, thereby limiting NLRP3 inflammasome activation and providing a crucial endogenous negative feedback mechanism against inflammation in obesity (77). IL-4 induces UCP1 expression in WAT, elevating basal metabolic rate (78).

Notably, obesity-induced immunometabolic imprinting within adipose tissue persists after weight loss and progressively deteriorates with weight cycling, and weight cycling has been proven to increase cardiometabolic diseases and disrupt glucose homeostasis in both human and animal models (79). Obesity itself and weight cycling both lead to adipose tissue inflammation and metabolic dysfunction: studies have revealed that weight cycling leads to impaired recovery of type 2 regulatory populations, sustained activation of antigen-presenting cells, T-cell exhaustion, and enhanced lipid handling in macrophages (80); even after weight loss, the increase in lipid-associated macrophages and memory T cells in WAT results in a more severe inflammatory state upon weight regain (79), which indicates that the immune system retains an “obesity memory” and this memory may contribute to the elevated inflammation and metabolic dysfunction associated with weight cycling. Compared with non-cycling obese mice, weight-cycling animals display impaired glucose tolerance, exacerbated adipose tissue insulin resistance, and increased pro-inflammatory T-cell infiltration (81). Human studies show positive correlations between weight cycling and increased central adipose tissue deposition, though its relationship with type 2 diabetes risk remains complex (82). This persistent and progressive phenotype provides a mechanistic explanation for how obesity-associated metabolic disturbances are sustained long after the initial insult and highlights the importance of sustained weight management for preventing obesity-related metabolic disease. Thus, by controlling their secretory profile and metabolic memory, macrophages form a crucial link between mitochondrial function, immune activity, and whole-body metabolic balance.

## 5 Microbiota reshapes ATMs

The gut microbiota and its metabolites are key environmental factors regulating host immune and metabolic homeostasis. Changes in gut microbiota composition strongly influence the development of obesity (83). Specifically, in obese conditions, gut microbiota may be dominated by potentially pro-inflammatory bacteria such as *Ruminococcus* or *Bacteroides* (84). Obesity also alters gut microbiota composition, increasing production of microbe-derived endotoxins (e.g., LPS) (85). Elevated circulating LPS activates innate immune pathways, fueling systemic inflammation and insulin resistance (86). These LPS-driven effects are further amplified by specific molecular mediators. For instance, a recent study identified the long non-coding RNA LncRNA29RIK as a key regulator of macrophage responses to LPS in the context of obesity (87). Using a murine model of high-fat diet-induced obesity, the investigators demonstrated that LncRNA29RIK expression in macrophages promotes LPS-mediated obesity sensitivity, meaning that it enhances the susceptibility of macrophages to LPS stimulation, leading to exacerbated inflammatory responses and worsened metabolic outcomes. Beyond direct LPS signaling, obesity-associated gut microbiota alterations also trigger metabolic changes in macrophages. These triggers increased GPAT3 expression, which leads to increased production of lysophosphatidic acid (LPA) and enhanced lipid droplet accumulation. This process activates the extracellular signal-regulated kinase (ERK) signaling pathway, ultimately increasing production of proinflammatory cytokines including TNF-α, IL-6, and IL-1β (88). TNF-α secretion derived from Kupffer cells also upregulates GPAT3, forming a continuous inflammatory cycle. GPAT3 deficiency limits LPA production and lipid droplet formation, resulting in reduced pro-inflammatory cytokine production and consequently anti-inflammatory effects (89). Thus, the microbiome-obesity link represents a promising new research direction, with macrophages potentially serving as key mediators. Recent research suggests that specific probiotics, prebiotics, and their metabolites could offer new ways to tackle obesity and related metabolic disorders by regulating the polarization and function of ATMs. Microbial metabolites directly participate in immune regulation (90). For example, L-ornithine produced by Lactobacillus can influence macrophage function through its downstream metabolic pathways. Polyamine metabolites produced during L-ornithine metabolism, such as spermine (SPM) and spermidine (SPD), play crucial regulatory roles in macrophage polarization. SPM curbs pro-inflammatory cytokine production by blocking NF-κB and Akt pathways. In contrast, SPD activates Src kinase and boosts IDO-1 levels, encouraging macrophages to adopt an immunosuppressive, IDO-1+ phenotype (90). Together, these actions alleviate tissue inflammation. Specific probiotic strains demonstrate potential to improve metabolism by modulating ATMs polarization. *Phocaeicola lactophilus Y01* promotes anti-inflammatory M2 polarization of ATMs by reshaping gut microbiota composition and its metabolic profile, thereby alleviating high-fat diet-induced obesity and metabolic syndrome (91). Similarly, the commensal bacterium *Faecalibacterium prausnitzii* promotes M2 macrophage polarization in both intestinal and adipose tissues, reversing obesity-associated immune dysregulation to alleviate glucose intolerance and weight gain (92). The probiotic *Escherichia coli Nissle 1917* improves insulin resistance in obese conditions by regulating the ubiquitination pathway of the RNF150/ELAVL1 protein (93). Furthermore, certain probiotics (e.g., *strain GB104*) emerge as promising dietary intervention candidates due to their capacity to stimulate glucagon-like peptide-1 (GLP-1) secretion. Probiotic strains such as *GB104*, which stimulate GLP-1 secretion, show promise as dietary interventions against obesity and metabolic disorders (94). *Lactobacillus acidophilus Y01* improves obesity-associated metabolic syndrome by mediating M1/M2 ATMs polarization through gut microbiota regulation. Overall, as a dietary supplement, *Lactobacillus acidophilus Y01* shows promising potential for obesity prevention and treatment (91). Symbiotic *Faecalibacterium* promoted polarization toward alternative activation M2, thereby reversing obesity-induced increases in gut-resident type 1 innate lymphoid cells. This resulted in improved glucose tolerance and reduced obesity, and weight gain (92). Dietary prebiotics indirectly regulate immunometabolism by encouraging the growth of beneficial bacteria. For example, *xylose (Xyl)* and its derivative treatments increase the abundance of beneficial bacteria such as *Lachnospiraceae NK4A136* and *Eubacterium xylanophilum* in the gut, while reducing obesity-associated genera (e.g., *Blautia*). This improved microbial composition correlates closely with reduced macrophage infiltration in adipose tissue, decreased expression of pro-inflammatory cytokines (e.g., TNF-α, IL-1β), and enhanced systemic glucose tolerance, indicating that prebiotic effects constitute one mechanism underpinning its metabolic benefits (95). Targeting the gut microbiota through supplementation with specific probiotics, prebiotics, or their beneficial metabolites can modulate the polarization state and inflammatory activity of ATM at multiple levels. This offers a promising nutritional immunology strategy for intervening in obesity-related adipose tissue inflammation and systemic metabolic disorders.

## 6 ATM-mediated metabolic disorders

ATMs play a central driving role in the pathological progression of obesity and related metabolic disorders. During obesity, adipose tissue macrophage numbers can surge by 40%, with their accumulation closely linked to worsening systemic metabolic syndrome (96). The function of macrophages is fundamentally tied to metabolic reprogramming. Together, these mechanisms create a complex network centered on adipose tissue macrophages. Targeting their reprogramming or interfering with their mediated communication may offer innovative therapeutic strategies for systematically improving obesity and related metabolic complications.

### 6.1 Type 2 diabetes mellitus

Type 2 diabetes mellitus(T2DM) is a chronic progressive metabolic disease that is closely associated with obesity and is characterized by insulin resistance, chronic low-grade inflammation, and progressive β-cell dysfunction (97). The relationship between obesity and type 2 diabetes is modulated by a complex network of endocrine and metabolic mechanisms. ATMs are central players in the development and progression of T2DM by driving and maintaining chronic low-grade inflammation in obese individuals (98). They release large amounts of mediators such as IL-1β, IL-6, TNF-α, NOS2, and the chemokine CCL2, directly interfering with insulin signaling and inducing lipolysis dysfunction, thereby promoting insulin resistance and the development of type 2 diabetes. Beyond local regulation in adipose tissue, adipose tissue modulates metabolic homeostasis in distal organs through the secretion of adipokines and non-coding RNAs. for example, adipocyte-derived miR-27a induces insulin resistance in C2C12 skeletal muscle cells by inhibiting PPARγ and its downstream genes associated with obesity development, which further expands the systemic damage of ATM-mediated insulin resistance (99). A chronic hyperglycemic environment further amplifies proinflammatory macrophage polarization through feedback activation of pathways including JNK, ERK, IL-4Rα-Tyk2-STAT6, and GLUT1-Akt-mTOR-AMPK, creating a vicious cycle (100).Several studies have shown that IL-4, IL-6 and TNF-α can enhance insulin resistance and type 2 diabetes by promoting GATA3 expression (101–103). In IR individuals, GATA3 expression is upregulated in adipose tissue and contributes to the induction of the inflammatory cytokine IL-6, which further exacerbates IR (104). Notably, the number of Pdpn+ macrophage subsets in perivascular adipose tissue (PVAT) is reduced in T2DM rats, which is confirmed in mice and humans (105); *in vitro* and *in vivo* studies have shown that Pdpn+ macrophages alleviate insulin resistance through the Pla2g2d-DHA/EPA-GPR120 pathway and regulate adipokine/cytokine expression in adipocytes, highlighting the specific regulatory role of PVAT-resident ATMs in T2DM progression (105). At the same time, multiple lines of evidence have shown that the inflammasome plays an important role in exacerbating obesity-related insulin resistance (106). Therefore, M1 polarization of ATMs in obese individuals is the core inflammatory bridge connecting obesity and T2DM. Clinical cohort studies have confirmed that high plasma levels of macrophage migration inhibitory factor are associated with poor long-term outcomes in T2DM patients (107), providing a potential clinical prognostic marker for T2DM. In addition, targeting ATM lipid metabolism and adipokine secretion is a promising therapeutic strategy: LRG reduces adipocyte size in WAT of DM mice, increases FGF21 secretion, ultimately inhibiting TG synthesis in macrophages and alleviating insulin resistance (108).

### 6.2 Metabolic-associated fatty liver disease

Metabolic-associated fatty liver disease (MASLD) is increasingly prevalent in obese patients, and its severity increases with obesity progression. Chronic overnutrition leads to an increase in the volume and number of adipocytes to accommodate the excess lipid burden (109), and this adipocyte dysfunction triggers endoplasmic reticulum stress and cell death, releasing FFAs, damage-associated molecular patterns (DAMPs), and other pro-inflammatory mediators (110). In obesity, M1-like ATMs secrete copious pro-inflammatory cytokines, particularly TNF-α, which potently stimulate adipocyte lipolysis via activation of the MAPK and NF-κB signaling pathways (111). This ATM-driven lipolysis, coupled with the intrinsic capacity of macrophages and their pro-inflammatory cytokines to dysregulate adipocyte lipolysis (112), leads to the uncontrolled release of FFAs from WAT into the systemic circulation (63). The resulting chronic FFA overload—termed “lipid spillover”—exceeds the liver’s capacity for fatty acid oxidation and export, promoting hepatic steatosis and the accumulation of lipotoxic species that disrupt hepatocellular metabolic homeostasis. Beyond direct lipotoxicity, steatotic hepatocytes actively propagate metabolic dysfunction: extracellular vesicles (EVs) released from steatotic hepatocytes induce insulin resistance in healthy hepatocytes and alter hepatic lipid and glucose metabolism, thereby amplifying MASLD progression (113). Clinical evidence in humans further validates the pathogenic link between WAT inflammation and MASLD: individuals with MASLD exhibit exacerbated WAT inflammation and increased infiltration of immune cells—most notably macrophages (114). In the adipose tissue of MASLD patients, M1-like macrophages marked by CD11c expression secrete TNF-α, IL-6, and ROS, which promote insulin resistance and systemic metabolic disorders. These pro-inflammatory ATMs exhibit metabolic reprogramming characterized by enhanced glycolysis and impaired OXPHOS, leading to the accumulation of TCA cycle intermediates that further modulate immune responses and amplify inflammation (70).

### 6.3 Atherosclerosis

The occurrence and development of atherosclerosis is the result of the interaction between cholesterol metabolism disorder and chronic inflammation (115, 116). As the central immune cells in atherosclerosis (AS), macrophages play pivotal roles in the initiation, progression, and rupture of atherosclerotic plaques (117). Notably, ATMs in WAT especially visceral WAT, mediate AS progression primarily through remote paracrine regulation and metabolic reprogramming, which differs from the *in-situ* inflammatory effects of vascular resident macrophages (118).Elevated plasma cholesterol, especially LDL-C, promotes the recruitment and infiltration of monocytes into the arterial wall and perivascular adipose tissue, which is polarized into pro-inflammatory M1 macrophages driven by IFN-γ (119, 120), while ATMs regulate this polarization process through multiple pathways.

First, adiponectin, an adipose tissue-derived protective adipokine, binds to its receptors AdipoR1 and AdipoR2 on macrophages to activate downstream signaling cascades (e.g., AMPK-PPAR γ pathway), inducing a shift toward an M2-like phenotype (121). This shift enhances macrophage cholesterol efflux and inhibits pro-inflammatory cytokine secretion, exerting atheroprotective effects. Notably, AdipoR1 and AdipoR2 exhibit subtle subtype-specific contributions. AdipoR1 primarily mediates fatty acid oxidation (FAO) to enhance metabolic flexibility, while AdipoR2 regulates PPARγ-dependent cholesterol efflux (121). Second, PVAT-derived extracellular vesicles (PVAT-EXOs) serve as key mediators of ATM-vascular crosstalk. PVAT-EXOs deliver miR-382-5p and bone morphogenetic protein 4 to macrophages, upregulating the expression of cholesterol efflux transporters ABCA1 and ABCG1 via PPARγ activation (122). Third, metabolic reprogramming of macrophages particularly glucose metabolic reprogramming plays a critical role in plaque inflammation. Pro-inflammatory polarization of macrophages (induced by ATM-derived TNF-α/IL-6 or oxLDL upregulates the expression of glycolytic enzymes (e.g., GLUT1, PKM2), shifting energy metabolism toward aerobic glycolysis (117). This metabolic switch leads to the accumulation of TCA cycle intermediates (citrate, fumarate, succinate) and promotes PKM2 dimerization and nuclear translocation (117). Additionally, methylglyoxal, a byproduct of glycolysis, reacts rapidly with proteins to form advanced glycation end products (AGEs), which are primarily concentrated in macrophages surrounding the plaque necrotic core (123). AGEs bind to their receptor RAGE on macrophages, activating the NF-κB pathway to amplify pro-inflammatory signaling and accelerate plaque instability (123, 124).

WAT plays a key role in this process. FFAs, adipokines and endoplasmic reticulum stress signals released by adipose tissue can activate JNK/NF-κB pathway, promote the expression of chemokines, and accelerate the infiltration of macrophages. The phenotype of macrophages also changed accordingly. These macrophages polarize toward M1-like phenotypes (e.g., CD11c+ macrophages) and aggregate around hypertrophic, dead adipocytes to form “coronal structures,” becoming persistent inflammatory signals (96). Clinical studies further confirm that ATM infiltration in visceral WAT correlates positively with carotid intima-media thickness and plaque vulnerability in AS patients (117), highlighting the central role of ATMs in linking WAT inflammation to AS.

### 6.4 Osteoarthritis

Osteoarthritis (OA) is a complex, age and obesity-associated degenerative joint disorder. Its rising global incidence parallels demographic aging and the escalating prevalence of obesity (125). As the most abundant cell type in the OA synovium predominantly localized within the synovial lining layer, macrophages play a central role in disease pathogenesis (125). Wood et al. reclassified OA into classical OA (cOA) and inflammatory OA (iOA) subsets: cOA is characterized by cartilage remodeling, while iOA is marked by prominent inflammatory and proliferative features (125). Insulin-like growth factor-binding protein 5 is overexpressed in cOA, which is associated with the negative regulation of inflammatory mediators (125). In contrast, obesity-driven OA closely aligned with the iOA subtype exhibits more severe cartilage destruction and higher levels of apoptotic cells (ACs) in the synovium of obese OA mice (126). Mechanistically, enhanced M1 polarization of ATMs in obese synovium reduces the secretion of growth arrest-specific 6, impairing macrophage efferocytosis of synovial ACs (126). Accumulated ACs release intracellular contents (e.g., damage-associated molecular patterns, DAMPs), further triggering immune responses and the release of pro-inflammatory cytokines such as TNF-α, IL-1β, and IL-6. These cytokines induce chondrocyte homeostatic dysfunction in obese OA patients, forming a “synovial inflammation-chondrocyte damage” positive feedback loop (127). In patients with end-stage OA and moderate-to-severe obesity, intra-articular adipose tissue exhibits pronounced pathological remodeling: hypertrophic adipocytes are markedly enriched; fibrosis is significantly exacerbated; TLR4 expression is upregulated; and PPARγ expression is downregulated relative to lean OA controls (128). Macrophages co-expressing CD11c and CD206 representing a transitional or metabolically activated phenotype—have emerged as pivotal mediators in OA pathogenesis. Adipokine dysregulation, particularly elevated leptin and reduced adiponectin levels, correlates strongly with OA severity (129). A skewed M1/M2 macrophage ratio characterized by M1 macrophage accumulation drives disease progression through sustained low-grade inflammation (130). Specifically, M1 macrophages amplify joint inflammation by secreting pro-inflammatory cytokines—including IL-1β, IL-6, and TNF-α—and by activating resident synovial and chondral cells. Mechanistically, the CDK5–PPARγ/NF-κB signaling axis exacerbates obesity-associated OA by coordinately promoting pro-inflammatory M1-like macrophage polarization and chondrocyte apoptosis (131). Collectively, these insights underscore macrophage reprogramming—targeting metabolic, phenotypic, and signaling nodes as a therapeutically promising strategy to mitigate OA progression and enhance cartilage repair.

## 7 Targeted therapy

Given that chronic, uncontrolled inflammation driven by ATMs plays a central role in the pathogenesis of obesity-associated metabolic disorders including insulin resistance, T2DM, MASLD, AS, and OA strategies that target ATM polarization, metabolic reprogramming, and pro-inflammatory activation represent highly promising therapeutic approaches. However, conventional small-molecule anti-inflammatory drugs are severely limited by poor WAT specificity, severe off-target effects, insufficient tissue penetration, and substantial interindividual variability (132). Systemic immunomodulation further carries inherent risks of excessive immune activation, disrupted tissue homeostasis, and uncontrolled systemic inflammation (133, 134). Therefore, precise, WAT−oriented interventions that selectively target ATMs while minimizing systemic side effects are urgently needed. In the following sections, we summarize current and emerging therapeutic strategies targeting ATM biology, with a focus on improving specificity, efficacy, and safety for the treatment of obesity-related metabolic diseases.

### 7.1 Targeting ATMs mitochondria

Mitochondria not only serve as the energy center of macrophages but also are the core hub for their immune metabolic reprogramming. Under obesity conditions, ATM mitochondrial dysfunction manifests as impaired OXPHOS, excessive production of mtROS, and defective mitochondrial autophagy. These changes collectively drive and maintain the pro-inflammatory M1-like phenotype. Therefore, targeting the repair of ATM mitochondrial function has become a highly promising therapeutic strategy (70, 135).

Restoring the OXPHOS level of ATM is a direct strategy to break its pro-inflammatory cycle. However, traditional anti-inflammatory drugs have poor targeting ability and can cause severe side effects (136). IR-61 is a small molecule with lipid-soluble cationic properties. A study found that IR-61 can specifically act on ATM mitochondria. In a diet-induced obesity mouse model, IR-61 upregulates the expression of mitochondrial complex subunits by activating the ROS/Akt/Acly signaling axis, thereby shifting the metabolic pattern of ATM from glycolysis to OXPHOS, significantly inhibiting the expression of M1-type pro-inflammatory markers and improving fat tissue inflammation and systemic insulin resistance, thereby ameliorating chronic inflammation and obesity-related diseases (137). Mitochondrial autophagy is a key quality control mechanism for clearing damaged mitochondria and maintaining mtROS homeostasis (138). To date, many studies have explored the role of mitochondrial autophagy in regulating mitochondrial quantity and adaptive regulation in response to changes in the internal and external environment (139). Restoring mitochondrial phagocytic balance and promoting the clearance of irreversibly damaged mitochondria may become a potential treatment strategy for various chronic diseases.

In bone marrow-specific knockout mice with Bnip3 deletion, high-fat diet-induced ATM pro-inflammatory polarization and glycolytic conversion were significantly exacerbated, suggesting that BNIP3-mediated mitochondrial autophagy is an endogenous protective mechanism that limits obesity-related fat tissue inflammation (140). Rosiglitazone, a peroxisome PPARγ agonist, restores BNIP3 expression in obese WAT, which in turn enhances mitochondrial bioenergetics capacity, mitochondrial membrane potential and mitigates mtROS accumulation (141).Various natural products have been shown to affect the ATM inflammatory state by regulating mitochondrial function, such as eicosapentaenoic acid, which reduces oxidative stress in LPS-stimulated macrophages by activating PPARα and inhibiting the NF-κB pathway. The fungal-derived metabolite rhamnocyclin exhibits a unique bidirectional regulatory effect, not only inhibiting adipocyte hypertrophy but also restoring mitochondrial oxygen consumption rate and mitochondrial membrane integrity in LPS-stimulated macrophages and inhibiting ROS production, thereby reducing the release of inflammatory mediators such as IL-6, TNF-α, and NO. In summary, whether it is regulating mitochondrial autophagy, removing excessive ROS, or using the new delivery system as a new frontier for intervening in obesity-related metabolic inflammation, current research is still mainly limited to the cellular and animal levels. Whether these strategies can be safely and effectively transferred to the human body still needs further verification.

### 7.2 Extracellular vesicles

Extracellular vesicles (EVs) constitute a heterogeneous group of cell-derived membrane structures, including exosomes and microvesicles (142). The transcriptome of EVs exhibits significant tissue specificity and directly participates in regulating susceptibility to obesity and related metabolic diseases (143, 144). For example, adipocyte-derived EVs (AdEVs) enrich multiple adipocyte-highly-expressed proteins such as adiponectin, FABP4, and lipoprotein-1, which can be distributed within the lumen or on the membrane surface of AdEVs (145). This makes EVs a key tool for studying obesity mechanisms and related complications, developing novel biomarkers, and identifying intervention targets. Levels of circulating EVs closely track obesity and related metabolic dysfunction, and they normalize after weight loss. This highlights their potential as dynamic biomarkers (146, 147). EVs can serve as carriers for bioactive molecules, such as macrophage migration inhibitory factor, rapidly inducing cellular responses by activating the ERK1/2 signaling pathway (147). Furthermore, under lipotoxic conditions, hepatocyte-derived small EVs (Hep-sEVs) can target pancreatic macrophages, inducing TLR4-dependent proinflammatory responses that impair insulin secretion function (148). miRNAs carried by EVs also play a crucial role in regulating obesity-related pathologies. Pre-adipocyte-derived EVs alleviate middle-aged obesity by delivering miR-145 mimics to suppress macrophage M1 polarization, a process involving regulation of the SELL–NF-κB signaling pathway (149). On the other hand, EVs from ATMs can either help or harm β-cell function through the miR-155–Mafb axis (150). Expanding understanding of extracellular vesicles, particularly their role as mediators within evolving intercellular communication networks linking adipose tissue to obesity-associated macrophages and tissues, presents potential therapeutic targets for obesity (113, 151). Although EVs show promise as natural therapeutic carriers, their complex biology presents challenges for systematically elucidating their functions and action pathways. This also hinders the establishment of a universal EV efficacy evaluation system (152).

### 7.3 Probiotic extracellular vesicles

**Figure 4:**
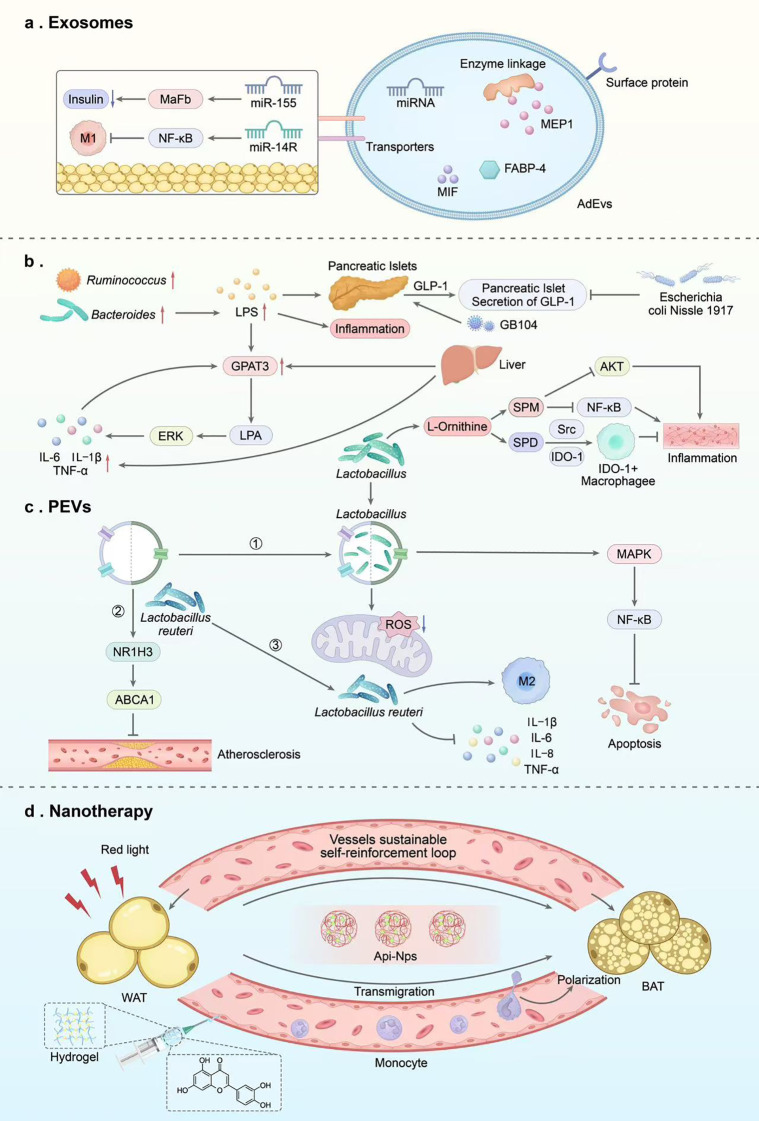
Novel therapeutic strategies targeting ATMs for obesity-related metabolic disorders. Emerging therapeutic strategies target ATMs via EVs, PEVs, and nanotechnology to reshape metabolic-immune homeostasis. **(a)** EV-mediated molecular delivery: AdEVs and pre-adipocyte-derived EVs carry bioactive molecules to regulate ATMs polarization (suppress M1, promote M2) and improve metabolic dysfunction. Circulating EVs serve as dynamic biomarkers for obesity and metabolic status. **(b)** Probiotic/metabolite modulation: Gut microbiota-derived metabolites and probiotics regulate ATMs polarization via NF-κB, Akt, and Src/IDO-1 pathways, alleviating inflammation. Probiotics like GB104 stimulate GLP-1 secretion to enhance metabolic control. **(c)** PEV-based therapy: PEVs derived from *Lactobacillus* and *Faecalibacterium prausnitzii* deliver bioactive cargo to modulate macrophage metabolism, promote M2 polarization, inhibit pro-inflammatory signaling (MAPK, NF-κB), and reduce ROS production, exerting anti-obesity, anti-atherosclerotic, and neuroprotective effects. **(d)** Nanotechnology-enabled precision intervention: Nanocarriers enable targeted drug delivery to ATMs and adipose tissue cells. Advanced nanosystems inhibit adipogenic precursor cell proliferation, reprogram ATMs phenotypes, and promote healthy adipose tissue remodeling via spatiotemporally controlled release and multi-effect synergy. ATMs, Adipose tissue macrophages; EVs, Extracellular Vesicles; PEVs, probiotic extracellular vesicles; NF-κB, Nuclear factor kappa-B; Akt, Protein kinase B; GLP-1, Glucagon-Like Peptide-1; MAPK, Mitogen-Activated Protein Kinases; ROS, Reactive oxygen species.

Probiotic EVs (PEVs) carry diverse bioactive compounds that can produce therapeutic effects like live bacteria. Through cytokine modulation and macrophage polarization, probiotic-derived EVs effectively regulate inflammation, offering a cell-free and safer alternative. We present them systematically in **Table 2** and **Figure 4**. *Faecalibacterium prausnitzii*-derived EVs alleviate chronic colitis and fibrosis by promoting fibrosis protection through a M2-like phenotype, achieved by suppressing glycolysis and oxidative phosphorylation, downregulating PPARγ and ABC transporters (153). In cardiometabolic diseases, *Lactobacillus*-derived EVs exhibit atherosclerosis protection by activating the NR1H3-ABCA1 axis, enhancing cholesterol efflux and promoting M2 macrophage polarization (154). In neuroinflammation, *Lactobacillus ramni*-derived EVs induce M2 microglial differentiation and reduce proinflammatory cytokines to alleviate perioperative neurocognitive dysfunction (155), while *Lactobacillus*-derived EVs prevent neuronal apoptosis after ischemic stroke via ROS scavenging and MAPK/NF-κB inhibition (156). Extracellular vesicles shed by macrophages co-cultured with Lactobacillus plantarum reduced adipocyte size in high-fat diet mice (157). These findings suggest that PEVs could serve as versatile platforms for reprogramming pathological macrophage metabolism. Future efforts should focus on optimizing production scalability, standardizing cargo composition, evaluating long-term safety, and conducting rigorous clinical trials.

**Table 2:** The role of probiotic extracellular vesicles regulating macrophage immune metabolism in metabolic diseases.

Names	Gram staining	Relevant mechanisms	Diseases	Results	Models	References
*Lactobacillus plantarum UJS001*	positive	The degradation pathway of lysine in amino acid metabolism	ulcerative colitis	↑M2 ↑IL-10 TGF-β↓ IL-6↓ TNF-α↓	UC mouse model	(158)
*Clostridium butyricum*	positive	SCAFs metabolism	ulcerative colitis	↑gut barrier related-proteins ↑colonic expression of M2 genes	UC mouse model	(159)
*Lactobacillus rhamnosus*	positive	promote lipid efflux and macrophage polarization	Atherosclerosis	↑NR1H3 ↑ABCA1 ↑lipid efflux from foamy macrophages ↑anti-inflammatory M2 phenotype	Atherosclerotic mice	(154)
*Escherichia coli Nissle 1917*	negative	bind with phosphorylated GSK3β	Osteoporosis	↑enzymatic stability and pre-osteoclasts permeability of FRATtide ↓ AKT/GSK3β/NFATc1	OP mouse model	(160)
*L. Plantarum*	positive	inhibited H2O2-induced oxidative damage	ulcerative colitis	↑macrophage to M2 type ↓the injury and shortening of colon tissue ↓proinflammatory cytokines ↑abundance of short-chain fatty acids	UC mouse model	(161)
*Lactobacillus paracasei*	positive	ER stress activation	ulcerative colitis	↓ IL-1α, IL-1β, IL-2, TNFα ↑IL-10 and TGFβ ↓ weight loss, ↑maintaining colon length, ↓ the disease activity index	UC mouse model	(162)
*Lactococcus lactis*	positive	Regulate the activation of M1 macrophages	inflammatory bowel disease	↓intestinal inflammation was partially dependent on EVs that contained miR-146b ↓the activation of M1 macrophages	murine IBD model	(163)
*Lactiplantibacillus plantarum No. 14*	positive	reduces adipocyte size in white adipose tissue	Obesity	↓adipocyte size ↓ adipogenesis ↓the mRNA levels of adipogenesis-related genes and insulin-induced glucose uptake	high-fat diet -induced obese mice	(157)

### 7.4 Nanomaterials: targeting ATMs

ATMs are key targets for regulating obesity-associated metabolic inflammation and homeostasis, yet their precise modulation remains challenging. Nanotechnology provides innovative tools for targeting ATMs precisely, thanks to its engineered delivery capabilities (164). Current nanostrategies primarily function at the following levels: 1. Enhanced drug delivery: For instance, nanoparticles loaded with curcumin or apigenin significantly improve the stability and targeting of these natural anti-inflammatory compounds, effectively modulating ATMs polarization and promoting adipose browning, thereby improving insulin sensitivity and reducing body weight (165, 166). 2. Precise modulation of the immune microenvironment: Advanced nanosystems enable multidimensional, precise regulation of adipose tissue cells. For instance, red-light-responsive bionic nanoparticles can deliver anti-inflammatory and browning-promoting drugs to M1 adipocytes and white adipocytes, respectively; while nanoparticles coated with macrophage membranes can deliver drug combinations and achieve a “self-reinforcing” targeting effect that improves with treatment (167, 168). 3. Specific Intervention of Adipogenic Precursor Cells: Nanocarriers can be engineered to selectively influence adipogenic precursor cells. Studies confirm that polyϵ-caprolactone nanoparticles loaded with disulfiram can specifically induce mitochondrial damage and apoptosis, potentially inhibiting adipose tissue proliferation (169). 4. Integrated “therapy-regeneration”: Cutting-edge nanobiomaterials (e.g., nanofiber hydrogel composites) transcend mere metabolic regulation by programmably modulating macrophage recruitment and phenotype, synergistically promoting angiogenesis and progenitor cell recruitment to guide healthy adipose tissue remodeling (170). Nanotechnology has pioneered highly promising new avenues for precisely regulating ATM and the adipose tissue immune microenvironment through efficient targeted delivery, spatiotemporally controlled release, and multi-effect synergistic interventions. Future research should aim to develop more subtype-specific targeting methods and move them toward clinical use to address therapeutic needs for metabolic diseases.

## 8 Conclusion and future prospect

A growing body of research highlights the crucial role of macrophage-mediated inflammatory states in obesity, which is closely linked to macrophage polarization and metabolic reprogramming.

This review synthesizes current insights into the metabolic state changes of macrophages during the progression of metabolic diseases, aiming to provide new perspectives for future research and therapeutic approaches. Additionally, we review prospective therapeutic strategies targeting macrophage immunometabolism, which may establish novel therapeutic pathways for treating obesity. Given the complexity of WAT pathophysiology, ATM immunometabolic research presents both opportunities and challenges. We need to explore how ATMs with complex or mixed phenotypes change and behave in different settings, such as different pathological states including obesity, aging, cancer, and even infection. Key questions remain unresolved: which microenvironmental factors drive critical ATM transitions, and how ATMs respond to these microenvironmental cues across different physiological or pathological states, particularly during shifts from metabolic adaptation to maladaptation. Tracking precisely where and when ATM changes occur during disease progression remains a challenge. Advancing translational research for targeted therapies: Existing intervention strategies are largely confined to cellular or animal studies; future efforts should validate the clinical relevance of novel targets using human samples. Developing highly specific delivery systems (e.g., ATM-targeted nanocarriers) to enhance drug efficacy and reduce off-target effects; conduct clinical trials of nutritional interventions like probiotics/prebiotics to clarify their efficacy and mechanisms in humans, accelerating translation from basic research to clinical application. The complexity of obesity-related diseases makes single-target interventions insufficient for achieving optimal outcomes. Future work should use a “metabolic-immunological microenvironment remodeling” approach to test combination therapies (e.g., mitochondrial-targeted drugs + probiotics, extracellular vesicle delivery systems + immunometabolic modulators). By synergistically regulating ATM function, gut microbiota, and systemic metabolic networks, these approaches aim to achieve precision and personalized treatment for obesity-related metabolic disorders.
